# Activity of Oritavancin and Its Synergy with Other Antibiotics against *Mycobacterium abscessus* Infection In Vitro and In Vivo

**DOI:** 10.3390/ijms22126346

**Published:** 2021-06-14

**Authors:** Gaoyan Wang, Jia Tang, Jiajia Feng, Wenqi Dong, Xinyu Huo, Hao Lu, Chenchen Wang, Wenjia Lu, Xiangru Wang, Huanchun Chen, Chen Tan

**Affiliations:** 1 State Key Laboratory of Agricultural Microbiology, College of Veterinary Medicine, Huazhong Agricultural University, Wuhan 430070, China; 97wgy@webmail.hzau.edu.cn (G.W.); tangjia_6@163.com (J.T.); 19fj@webmail.hzau.edu.cn (J.F.); 13137204926@163.com (W.D.); 2020302110145@webmail.hzau.edu.cn (X.H.); sdluhao521@163.com (H.L.); 2018302110164@webmail.hzau.edu.cn (C.W.); hzaulwj1995@163.com (W.L.); wangxr228@mail.hzau.edu.cn (X.W.); chenhch@mail.hzau.edu.cn (H.C.); 2 Key Laboratory of Preventive Veterinary Medicine in Hubei Province, Wuhan 430070, China; 3 International Research Center for Animal Disease, Ministry of Science and Technology of the People’s Republic of China, Wuhan 430070, China

**Keywords:** *Mycobacterium abscessus*, oritavancin, synergy, immunosuppressive mice model

## Abstract

Background: Pulmonary disease caused by *Mycobacterium abscessus* (*M. abscessus*) spreads around the world, and this disease is extremely difficult to treat due to intrinsic and acquired resistance of the pathogen to many approved antibiotics. *M. abscessus* is regarded as one of the most drug-resistant mycobacteria, with very limited therapeutic options. Methods: Whole-cell growth inhibition assays was performed to screen and identify novel inhibitors. The IC_50_ of the target compounds were tested against THP-1 cells was determined to calculate the selectivity index, and then time–kill kinetics assay was performed against *M. abscessus*. Subsequently, the synergy of oritavancin with other antibiotics was evaluated by using checkerboard method. Finally, in vivo efficacy was determined in an immunosuppressive murine model simulating *M. abscessus* infection. Results: We have identified oritavancin as a potential agent against *M. abscessus*. Oritavancin exhibited time-concentration dependent bactericidal activity against *M. abscessus* and it also displayed synergy with clarithromycin, tigecycline, cefoxitin, moxifloxacin, and meropenem in vitro. Additionally, oritavancin had bactericidal effect on intracellular *M. abscessus*. Oritavancin significantly reduced bacterial load in lung when it was used alone or in combination with cefoxitin and meropenem. Conclusions: Our in vitro and in vivo assay results indicated that oritavancin may be a viable treatment option against *M. abscessus* infection.

## 1. Introduction

*M. abscessus,* belonging to the rapidly growing mycobacteria, is an environmental pathogen that can cause chronic pulmonary infections in humans, especially in immune-compromised individuals, such as bronchiectasis and cystic fibrosis (CF) [1,2]. Additionally, the infection with *M. abscessus* results in accelerated damage to lungs, and it has been considered as a contraindication to lung transplantation [3,4]. Unfortunately, the inherent resistance of *M. abscessus* to some antibiotics, including several antituberculosis drugs, makes it one of the most resistant pathogens, posing a major threat to public health [5]. Except intrinsic resistance, adaptive and acquired resistances are also one of the important reasons why treatment of *M. abscessus* infection remains extremely difficult [6]. The US Cystic Fibrosis Foundation and the European Cystic Fibrosis Society have recommended several antibiotics such as macrolides, aminoglycosides, β-lactam, fluoroquinolones, and tetracyclines [7,8]. However, *M. abscessus* tends to develop acquired resistance to these antibiotics [9]. Besides antibiotics therapy, Rebekah et al. (2019) reported a phage therapy consisting of a cocktail of three engineered phages which was used to treat *M. abscessus* infection in a teenager afflicted with CF after bilateral lung transplantation [10].

Until now, there has been no official standard drug regimen for the treatment of *M. abscessus* infection. According to the latest guidelines of the American Thoracic Society and the Infectious Diseases Society of America (ATS/IDSA), it is suggested to use at least three active drugs among patients without macrolide resistance, and at least four active drugs among patients with macrolide resistance in the initial treatment phase (amikacin, imipenem (or cefoxitin), tigecycline, and azithromycin (clarithromycin), clofazimine, linezolid); and at least 2–3 active drugs for the continuation therapy phase (azithromycin (clarithromycin), clofazimine, linezolid, amikacin) [11,12]. However, current therapeutic regimens are usually accompanied by severe side effects [5]. Therefore, there is urgent medical need for more effective antibiotics treatment regimens to be developed for control this global threat.

Surprisingly, there are many compounds in clinical trials against *M. tuberculosis*, but few against *M. abscessus* [6]. As we know, development of novel antimicrobial drugs requires a complex and time-consuming verification process. Repurposed drugs may be a viable pathway for developing new treatment regimens. In the process of screening a library of drugs approved by the Food and Drug Administration for use against *M. abscessus*, we found that the lipoglycopeptide oritavancin possessed bacteriostatic activity against *M. abscessus*. Recently, several studies have reported that vancomycin and oritavancin show similar bacteriostatic activity against the clinically isolated *M. abscessus* complex, and there was synergy of vancomycin and clarithromycin against *M. abscessus* complex in vitro [13,14]. However, the therapeutic activity of oritavancin and vancomycin against *M. abscessus* in vivo remains largely unknown. In this study, we demonstrated that oritavancin exhibited activity against *M. abscessus,* both in vitro and intracellular. Oritavancin and clinically approved antibiotics displayed synergism in vitro. Moreover, oritavancin showed significant activity against *M. abscessus* in vivo when it was used alone or combined with cefoxitin and meropenem. Hence, our work may provide a viable therapeutic regimen against *M. abscessus* infection.

## 2. Results

### 2.1. Activity of Oritavancin against M. abscessus and M. tuberculosis

**Figure 1:**
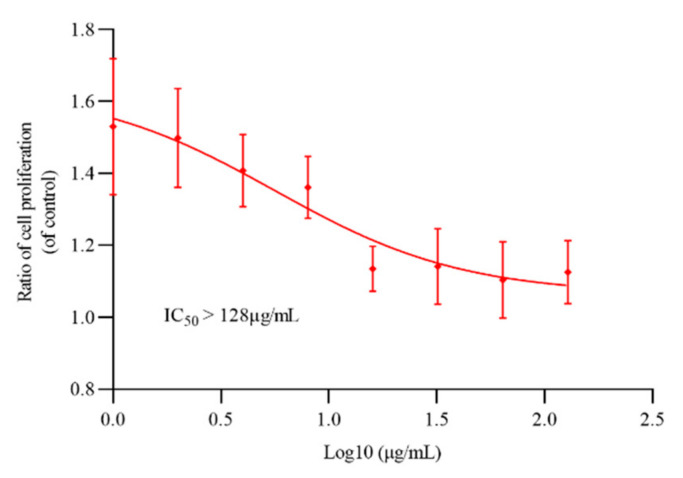
The cytotoxicity of oritavancin against THP-1 cells. The THP-1 cells were incubated with different concentrations of oritavancin for 72 h to determine its cytotoxicity. Data are expressed as the mean ± standard deviation (SD) of triplicates for each concentration.

*M. bovis* BCG and *M. smegmatis* mc^2^155 were commonly used as model strains to evaluate the antituberculosis activity of novel compounds. To determine the activity of oritavancin against *M. abscessus* and *M. tuberculosis*, the sensitivity of reference strains to oritavancin was tested by REMA (resazurin microtiter assay) method. As shown in Table 1, oritavancin not only had an antibacterial effect on *M. tuberculosis*, but also exhibited activity against highly drug-resistant *M. abscessus*. The determination results of cytotoxicity of oritavancin against THP-1 macrophages indicated that IC_50_ > 128 μg/mL (Figure 1) and selectivity index (SI) >16 (for *M. abscessus*) and SI > 256 (for *M. tuberculosis* H37Rv) (SI = IC_50_/MIC) suggesting that oritavancin possessed a high selectivity to *M. abscessus* and *M. tuberculosis*.

### 2.2. Bactericidal Activity of Oritavancin against M. abscessus and M. bovis BCG In Vitro and Intracellular

**Figure 2:**
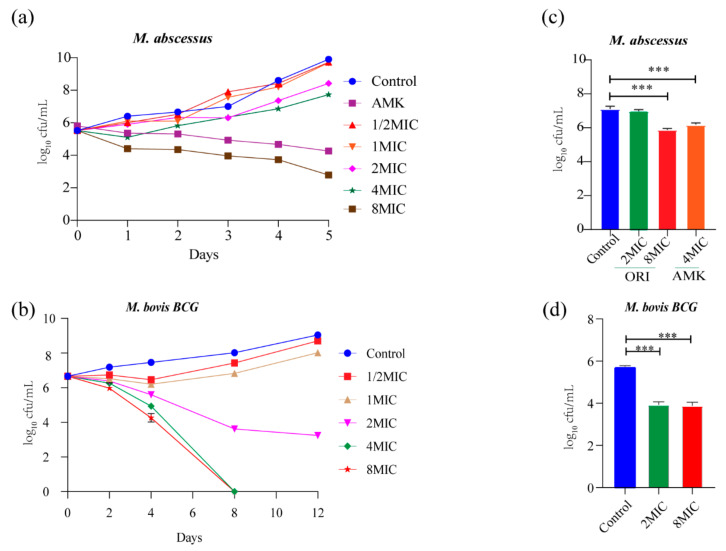
In vitro and intracellular bactericidal activity of oritavancin against *M. abscessus* and *M. bovis* BCG. Bactericidal activity of oritavancin against *M. abscessus* (**a**) and *M. bovis* BCG (**b**) was determined with different concentrations of oritavancin. And effect of oritavancin on *M. abscessus* (**c**) and *M. bovis* BCG (**d**) of viability in THP-1 cells was determined on 72 h after treatment with 2 × MIC and 8 × MIC oritavancin. Data are expressed as the mean ± standard deviation (SD) of triplicates for each concentration. The error bar is smaller than the symbol size and not showed. *** *p* < 0.001. ORI: oritavancin, AMK: amikacin.

The potency of oritavancin (1/2 × MIC~8 × MIC) killing *M. abscessus* and *M. bovis* BCG was evaluated. As shown in Figure 2a, 7.1 log10 CFU and 5.6 log10 CFU reduction was observed at day 5 post treatment with oritavancin at 8×MIC and amikacin at 4 × MIC against *M. abscessus*, which was compared with untreated control. Moreover, no viable cells were recovered after 5-day treatments, suggesting limited probability of generating drug resistance. The 8-day treatment with oritavancin against *M. bovis* BCG at 4 × MIC caused 8 log10 CFU reduction (Figure 2b). In addition, the ability of oritavancin to clear intracellular infection of *M. abscessus* and *M. bovis* BCG was determined. The results indicated that at 8 × MIC, oritavancin reduced the intracellular *M. abscessus* by 1.2 log10 CFU, whereas amikacin at 4 × MIC reduced the cells by 0.9 log10 CFU as compared with the control (Figure 2c). The oritavancin (at 8×MIC) against *M. bovis* BCG resulted in 1.8 log10 CFU reduction (Figure 2d). Taken together, our data indicate that oritavancin exhibited significant killing activity against *M. abscessus* and *M. tuberculosis,* in vitro and intracellular.

### 2.3. Interactions with Other Clinically Utilized Antibiotics

**Figure 3:**
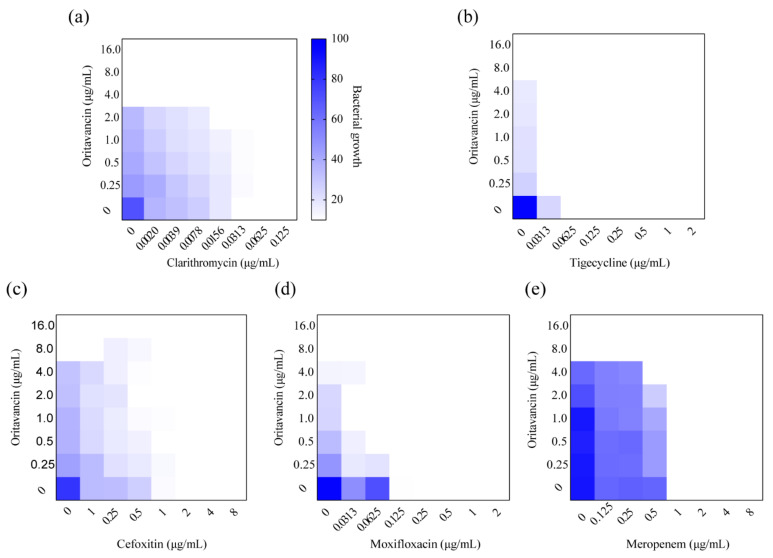
Synergism of oritavancin with (**a**) clarithromycin, (**b**) tigecycline, (**c**) cefoxitin, (**d**) moxifloxacin, and (**e**) meropenem against *M. abscessus*. Dark blue regions indicate high bacterial growth with low inhibition rate. The MICs of oritavancin, clarithromycin, tigecycline, cefoxitin, moxifloxacin, and meropenem against *M. abscessus* were 8.0 μg/mL, 0.0625 μg/mL, 1.0 μg/mL, 4.0 μg/mL, 1.0 μg/mL, and 4.0 μg/mL, respectively. Data are expressed as the mean OD_600_ of two biological replicates. The FICI ≤ 0.05 is defined as synergy.

Single-drug treatments against *M. abscessus* are limited and rapidly led to drug resistance, therefore, drug combinations are necessary in the treatment of *M. abscessus* infection [15]. The combined effect of oritavancin and antibiotics utilized for clinical treatment was examined in vitro against *M. abscessus* by a checkerboard method. As shown in Table 2 and Figure 3, oritavancin exhibited a synergy with clarithromycin, tigecycline, and cefoxitin with ∑FIC values of 0.5, 0.141, and 0.5, respectively (Figure 3a–c). In addition, the combination of oritavancin with moxifloxacin and clarithromycin displayed synergy with ∑FIC values of 0.5 and 0.226 (Figure 3d,e). More importantly, no antagonism between oritavancin and these antibiotics was observed, suggesting that oritavancin possessed the potential to be utilized for multidrug therapy treat *M. abscessus* infections.

### 2.4. M. abscessus-Killing Effect of Oritavancin in Combination with Clarithromycin, Cefoxitin, Moxifloxacin, Tigecycline, or Meropenem

**Figure 4:**
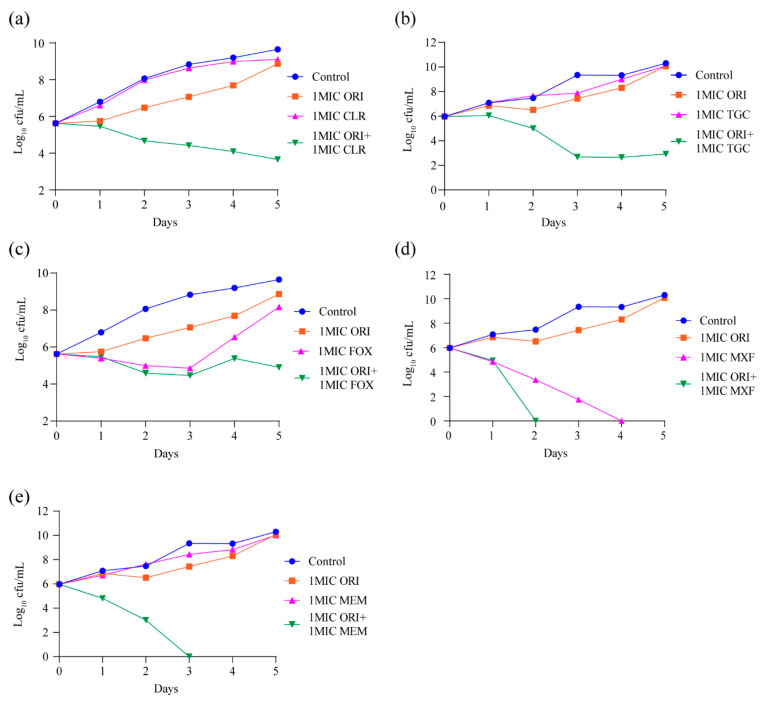
Oritavancin significantly potentiates (**a**) 1 × MIC clarithromycin, (**b**) 1 × MIC tigecycline, (**c**) 1 × MIC cefoxitin, (**d**) 1 × MIC moxifloxacin, and (**e**) 1 × MIC meropenem bactericidal effect against *M. abscessus* in vitro. Data are represented the mean ± standard deviation (SD) of triplicates for each concentration. The error bar is smaller than the symbol size and not showed. ORI, oritavancin; CLR, clarithromycin; TGC, tigecycline; FOX, cefoxitin; MXF, moxifloxacin; MEM, meropenem.

Our in vitro MIC testing confirmed that oritavancin could synergize with other approved antibiotics. The result of time–kill kinetics assay of oritavancin against *M. abscessus* showed that oritavancin has no bactericidal effect at 1 × MIC (Figure 2a). Therefore, we further explored whether the combination of oritavancin respectively with clarithromycin, cefoxitin, moxifloxacin, tigecycline, or meropenem at 1 × MIC could improve the bactericidal activities of both antibiotics. As shown in Figure 4a,b, when oritavancin was in combination with clarithromycin or tigecycline, a 6 log10 and 7.4 log10 CFU decrease was observed on day 5. In the case of the treatment with cefoxitin alone at 1 × MIC, bactericidal effect was observed over the first 3 days of exposure, followed by an increasing CFUs afterwards. The treatment with moxifloxacin alone at 1 × MIC exhibited a strong bactericidal effect on day 4. The time–kill kinetics assay results of oritavancin at 1 × MIC in combination of cefoxitin or moxifloxacin indicated that combined bactericidal effect of two antibiotics was superior to that of single one, Figure 4c,d. Furthermore, we found that treatment with meropenem alone at 1 × MIC had weak inhibitory activity, and that the combination of oritavancin with meropenem at 1 × MIC resulted in complete killing on day 3 with no viable bacteria recovered after 2-day culture (Figure 4e). Thus, these results indicated the potential of oritavancin to be used as an adjuvant drug against *M. abscessus* infection.

### 2.5. Activity of Oritavancin against M. abscessus Infection in Immunosuppressive Mouse Model

**Figure 5:**
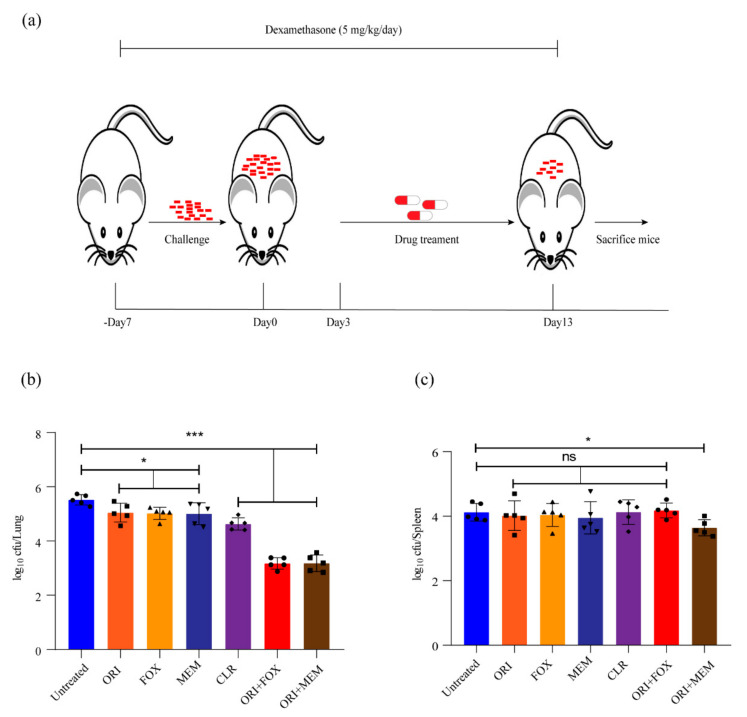
Evaluation of in vivo efficacy of oritavancin alone and its combination with cefoxitin or meropenem against *M. abscessus* in immunosuppressive mouse model. (**a**) Schematic diagram of mouse infection and drug treatment. –Day 7, 7 days before infection; Day 0, the day when challenge begins; Day 3, the day when infection ends and drug treatment starts; Day 13, the day when therapy ends and mice are sacrificed. The log10 cfu/mL indicates bacterial load in lungs (**b**) and spleen (**c**) of infected mice after treatment with 50 mg/kg oritavancin (ORI), 200 mg/kg cefoxitin (FOX), and 100 mg/kg meropenem (MEM) alone or in combination. The treatment with 100 mg/kg clarithromycin (CLR) serves as positive control. * *p* < 0.05, *** *p* < 0.001; ns, not significant.

Several β-lactams have been approved for the treatment of infection caused by *M. abscessus* in the United States and Europe [8,16,17]. Our results indicated in vitro synergy of oritavancin with cefoxitin, or meropenem against *M. abscessus*. The combined effect of these antibiotics against *M. abscessus* infection was further investigated in vivo. Thus, we utilized an immunosuppressive murine model to evaluate the therapeutic efficacy of these antibiotics in vivo (Figure 5a). As shown in Figure 5b, treatment with oritavancin (at 50 mg/kg), cefoxitin (at 200 mg/kg), and meropenem (at 100 mg/kg) used caused an approximate reduction of 0.5 log10 CFU in lungs, and clarithromycin (at 100 mg/kg) alone led to a reduction of 0.9 log10 CFU, whereas oritavancin in combination with cefoxitin or meropenem resulted in reductions of 1.4 log10 CFU and 1.3 log10 CFU in lungs, respectively. In addition, a reduction of only 0.6 log10 CFU in spleen was observed when oritavancin was combined with meropenem (Figure 5c). These results revealed that oritavancin had an effect similar to cefoxitin or meropenem in reducing bacterial load in the lungs of infected mice, and that the combination of oritavancin with cefoxitin or meropenem could significantly enhance the clearance of *M. abscessus* in the lungs.

## 3. Discussion

*M. abscessus* is a serious threat to human health which is naturally resistant to a broad range of antibiotics, and there is a lack of new active molecules. So far, treatment of *M. abscessus* infection has been particularly difficult, with a low cure rate [6,18]. Clarithromycin-based therapy regimens are less effective, and clarithromycin resistance in some isolates thus leads to treatment failure [19]. Therefore, finding novel antibacterial drugs to control *M. abscessus* infection is sorely needed. Currently, several different drug discovery approaches have been conducted, such as whole-cell screening, new combination studies that cause synergistic effects with already existing drugs or drug repositioning studies with old drugs [19]. Furthermore, conventional drug in new use offers a viable strategy for the clinical therapy of *M. abscessus* infection by screening anti-*Mycobacterium abscessus* drugs from an approved drug library. In this study, we found that oritavancin exhibited mild inhibition against *M. abscessus*. Oritavancin, as a new glycopeptide antibiotic, is active against several Gram-positive bacteria and pharmacokinetics characterized by extensive tissue distribution and a long terminal half-life, which may be an attractive option for the treatment of *M. abscessus* infection. It is effective in treating drug-resistant bacteria and clinical isolates [14]. Thus, the efficacy of oritavancin in the treatment of *M. abscessus* infections remains to be determined.

*M. abscessus* infections generally require treatment with multidrug combinations [20], oritavancin is active against several Gram-positive bacteria, which may be an attractive option for the treatment of *M. abscessus* infection. In the present study, oritavancin was active against *M. abscessus*, the MIC was 8 μg/mL. We also found that oritavancin was effective in inhibiting the growth of intracellular *M. abscessus* in vitro at concentrations effective in inhibiting organisms growing extracellularly. However, the results of cytotoxicity showed that oritavancin has mild inhibition to THP-1 cells at a concentration of 64 μg/mL, which may interfere with the bactericidal effect of oritavancin on intracellular bacteria to a certain extent. We may be able to evaluate the pharmacological activity in other macrophages in future experiments, such as RAW264.7, J774A.1, etc. More importantly, the therapeutic activity of oritavancin was also evaluated in an immunosuppressive mouse model. Interestingly, oritavancin, with a quarter of the dose of cefoxitin or a half of the dose of meropenem, exhibited a similar activity to cefoxitin and meropenem in reducing bacterial load in the lungs of infected mice. Clarithromycin, the positive control, used alone showed better effectivity than the other three antibiotics (oritavancin, cefoxitin, and meropenem). However, when oritavancin was combined with cefoxitin or meropenem, it exhibited better bactericidal effect than clarithromycin. Even so, there is a flaw in our animal experiment. In future experiments, it is necessary to conduct further experiments in the mouse model to probe the synergistic effect of oritavancin with other known antibiotics at higher doses. As we know, glycopeptide antibiotics will damage the cell wall synthesis, thus they may facilitate drug penetration and enhance drug activity [21]. However, the ability of oritavancin is not absolute. The results of the combination showed that oritavancin only exhibited a synergism with clarithromycin, cefoxitin, meropenem, moxifloxacin, and tigecycline. To our surprise, when oritavancin was combined with cefoxitin and meropenem, such an effect was also observed in an immunosuppressive mouse model against *M. abscessus* infection. As far as we know, this is the first report of the combination between oritavancin and two β- lactam antibiotics (cefoxitin and meropenem) against *M. abscessus* infection in vivo. Oritavancin exhibits single-use bactericidal activity and simultaneously enhances bacterial clearance, acting as an adjuvant. Nevertheless, extreme caution should be taken regarding the coadministration of oritavancin with other antibiotics in the clinical setting.

Data obtained in a phase III clinical trial, evaluating the effect of oritavancin as a targeted antibiotic for the treatment of patients with acute bacterial skin and skin structure infections, showed that an 138 μg/mL maximum plasma concentration was safely attained and well tolerated by patients (*n* = 297) administered with a single 1200 mg dose [22]. The plasma concentration is higher than the MIC of oritavancin against *M. abscessus* in vitro determined in this study, which suggests that an effective dose can be achieved for the treatment of clinical *M. abscessus* infections. Even so, the clinical application of oritavancin required to be further determined.

Moreover, one important factor to consider is that oritavancin is administered intravenously [23]. This will be a limitation of using oritavancin considering the long clinical therapeutics duration of *M. abscessus* infections. Intravenous administration is a main concern associated with oritavancin, making it difficult to treat chronic diseases. Thus, modifying the drug-delivery mode may be necessary to consider. For example, Wilcox et al. reported that oritavancin, administered orally, exhibits significant activity against CDI in a hamster model in a dose-dependent manner [24]. Moreover, future study is suggested to provide the data of the antibacterial activity of oritavancin against clinical *M. abscessus* isolates. In conclusion, this study indicates that oritavancin exhibits activity against *M. abscessus* in vitro and in vivo, and it does not antagonize other most frequently used antibiotics for treating *M. abscessus* infections, suggesting the potential of oritavancin as a clinical drug for treating *M. abscessus* lung disease.

## 4. Materials and Methods

### 4.1. Antimicrobial Agents

Oritavancin diphosphate and bedaquiline were obtained from TargetMol (Shanghai, China). The others antibiotics were purchased from Sigma-Aldrich (Shanghai, China). All the compounds mentioned above were diluted in accordance with the recommendations of the manufacturers, divided into aliquots, and stored at −20 °C.

### 4.2. Bacterial Strains, Cell Line, and Culture Conditions

*M. abscessus* ATCC19977, *M. tuberculosis* H37Rv ATCC27294, *M. tuberculosis* H37Ra ATCC25177, *M. bovis* ATCC19210, *M. bovis* BCG ATCC35737 (*M. bovis* BCG), and *M. smegmatis* mc^2^155 ATCC700044 were propagated in Middlebrook 7H9 broth (BD, New York, NJ, USA) supplemented with 10% OADC (BD, New York, NJ, USA), 0.2% glycerol (Sigma, Saint Louis, MO, USA), and 0.05% Tween 80 (Amresco, Houston, TX, USA) or on 7H11 agar plates supplemented with 0.5% glycerol and 10% OADC. THP-1 macrophages (ATCC TIB-202) were cultured in RPMI-1640 medium (Gibco, Shanghai, China) supplemented with 10% fetal bovine serum (Gibco, Waltham, MA, USA) at 37 °C with 5% CO_2_.

### 4.3. Mouse Experiments

Animal experiments were performed on 6-week-old female BALB/c mice purchased from the Experimental Animal Center, Huazhong Agricultural University.

### 4.4. Determination of Minimum Inhibitory Concentration (MIC) and Minimum Bactericidal Concentration (MBC)

The MICs were determined using the previously described resazurin reduction microplate assay (REMA) method, with slight modifications [25]. Briefly, logarithmic bacteria were diluted to an OD_600_ of 0.01. Two-fold serial dilutions of test compounds were performed in 96-well microplates. The MIC of each single antibiotic was tested in the panel with final drug concentrations ranging from 0.0625 to 128 μg/mL. The microplates were added with 30 µL of 0.01% resazurin solution per well and incubated for 24 h at 37 °C. A color change from blue to pink indicated bacterial growth. The minimum drug concentration that could prevent the color change was defined as MIC. MIC was determined at day 3 (for *M. abscessus* and *M. smegmatis*) and day 7 (for *M. tuberculosis*) post antibiotics incubation; 100 μL of the inoculum from each well were 10-fold serially diluted and plated onto 7H11 agar plates to count the colony-forming units (CFUs). The minimum drug concentration that kills 99.9% of bacteria was defined as the MBC. The experiments were performed in triplicate.

### 4.5. Cytotoxicity Assay

Cytotoxicity was evaluated with THP-1 cells using the WST-1 (Beyotime, Shanghai, China) assay. Cells were seeded into a 96-well microplate and incubated at 37 °C with 5% CO_2_ for 12 h. Different concentrations of oritavancin were added and incubated for 72 h. The 10 μL WST-1 solution was added into each well, and the microplate was incubated for another 2 h. Thereafter, the OD_450_ was monitored using a fluorescence microplate reader for calculating the 50% inhibitory concentration (IC_50_). The experiment was performed in triplicate.

### 4.6. Intracellular Anti-Mycobacterial Activity

THP-1 cells were seeded into 24-well plates and incubated with a final concentration of 100 nM phorbol 12-myristate 13-acetate (PMA, AbMole, Shanghai, China) for 48 h to induce cell differentiation into macrophages. Cells were infected with *M. abscessus* at a MOI (multiplicity of infection) 4:1(cell: bacterium) for 1 h or *M. bovis* BCG at a MOI of 1: 20 (cell: bacterium) for 4 h. The extracellular bacteria were removed by washing three times with PBS. Oritavancin was added to the corresponding well at 2 × MIC and 8 × MIC and 4 × MIC of amikacin was also added to the well as a positive control. After 72 h, the cell lysates were plated onto 7H11 agar plates to count CFUs. The experiments were performed in triplicate.

### 4.7. Time–Concentration-Dependent Killing Assay

Time–concentration-dependent killing abilities of oritavancin was determined as pre viously described [26]. Briefly, *M. abscessus* or *M. bovis* BCG with OD_600_ of 0.01 were cultured in 7H9 medium containing a range of 1/2 × MIC to 8 × MIC oritavancin. Amikacin, the positive control, was added to *M. abscessus* culture with OD_600_ of 0.01 to reach a final concentration of 4 × MIC. *M. abscessus* was cultured for 5 days and *M. bovis* BCG for 12 days at 37 °C. At the indicated time, 100 μL culture was serially diluted and plated onto 7H11 agar plates to count the CFUs.

For the combination of *M. abscessus* kill kinetic assays, oritavancin and its combinational antibiotics were added into 7H9 medium containing a final OD_600_ 0.01 of *M. abscessus* to reach a final concentration of 1 × MIC. As described above, the cultures were incubated for 5 days at 37 °C. During the 5-day incubation, cultures were serially diluted each day, and plated onto 7H11 agar plates. Subsequently, the resultant agar plates were incubated for 3 days at 37 °C, CFUs were counted. The experiments were performed in triplicate.

### 4.8. Synergy Assay

The synergic effect of oritavancin and other antibiotics against *M. abscessus* was determined using a checkerboard assay as described previously [27]. Briefly, a 50 μL of serial dilution of oritavancin was added along the ordinate, and an equal volume of serial dilution of other antibiotics were added along the abscissa into 96-well microplate. The logarithmic phase of *M. abscessus* was diluted to an OD_600_ of 0.01 and then added to each well. Subsequently, microplates were incubated at 37 °C for 3 days. After 3-day incubation, the OD_600_ of each well was measured with a Fluostar Omega microplate reader (SPARK 10M, TECAN, Männedorf, Switzerland). The inhibition of 90% of bacterial growth was defined as synergy. The FICI was calculated using the formula:

FICI = FIC_A_ (MIC of drug A combination/MIC of drug A alone) + FIC_B_ (MIC of drug B combination/MIC of drug B alone), FICI ≤ 0.5 indicated synergy; 0.5 < FICI ≤ 4 indicated no interaction; and FICI > 4 indicated antagonism. The experiments were performed in duplicate.

### 4.9. Bacterial Load Experiments

Several studies have demonstrated that immunocompetent mice can gradually clear the infection of *M. abscessus*, whereas certain immunodeficient mice can maintain chronic infection in vivo [28,29]. Maggioncalda et al. reported that dexamethasone treatment can suppress the immune response of mice to sustain *M. abscessus* infection [28]. To obtain adequate immunosuppression and avoid interfering with the therapeutic effect of antibiotics in vivo by the mice’s immune clearance, we treated the mice with dexamethasone to increase their susceptibility to an initial infection. Briefly, 6-week-old female BALB/c were randomly divided into seven groups (5 mice per group). Dexamethasone was administered by subcutaneous injection at 5 mg/kg/day (6 days per week), as previously described [30,31]. The administration of dexamethasone was initiated 1 week before infection and continued throughout the experiment. All mice were infected with 10^6^ CFU *M. abscessus* via intravenous injection. From day 3 after infection, the infected mice were treated daily with antibiotics for 10 days. Clarithromycin was prepared in 10% hydroxyethyl-β- cyclodextrin and administered at a dose of 100 mg/kg by oral gavage to the mice as positive control. Oritavancin, cefoxitin, and meropenem were administered via subcutaneous injection at the doses of 50 mg/kg, 200 mg/kg, and 100 mg/kg, respectively. The doses of clarithromycin (100 mg/kg), cefoxitin (200 mg/kg), and meropenem (100 mg/kg) were described previously [29,30,32]. Then, the dose of oritavancin (50 mg/kg) was chosen, based on pharmacokinetic studies of oritavancin in mice; the peak plasma concentration of oritavancin was 228.83 μg/mL when oritavancin was intravenous at dose 20 mg/kg of body weight [33]. The serum concentration is higher than the MBC of oritavancin against *M. abscessus* in vitro determined here. Oritavancin, cefoxitin, meropenem, and clarithromycin were used for single-drug treatment. The drug combination treatment was as follows: oritavancin, cefoxitin, oritavancin and meropenem. The untreated group was treated with PBS. All the mice were sacrificed on day 10 after drug treatment, lungs and spleen were removed and homogenized. Homogenates were plated onto 7H11 agar plates to calculate CFUs for determining the bacterial loads of these organs.

### 4.10. Ethical Approval

The use of mice for infection experiment (HZAUMO-2021-0013) was approved by the Tab of Animal Experimental Ethical Inspection of Laboratory Animal Centre, Huazhong Agriculture University.

### 4.11. Statistical Analysis

For all experiments, a Student’s *t*-test was used to evaluate differences between treated and control group and GraphPad Prism 8.0.1 software (GraphPad Software Inc., San Diego, CA, USA) was used to analyze data. Error bars indicate the standard deviation (SD) within the group. *p* ≤ 0.05 was considered to be significant.

## Figures and Tables

**Table 1:** MICs and MBCs of oritavancin against *M. abscessus* and *M. tuberculosis*.

	Concentration (μg/mL)	
Strains	MIC	MBC
*M. abscessus*	8	64
*M. tuberculosis* H37Rv	0.125	0.5
*M. tuberculosis* H37Ra	2	8
*M. bovis*	0.125	0.5
*M. bovis* BCG	0.5	1
*M. smegmatis* mc^2^155	8	32

**Table 2:** Synergy of oritavancin and approved antibiotics.

	MIC (μg/mL)				
Drug	Alone	Combination	FIC	∑FIC	Remarks
Oritavancin	8	2.0	0.25		
Clarithromycin	0.0625	0.0156	0.25	0.5	Synergism
Oritavancin	8	0.125	0.0156		
Tigecycline	1	0.125	0.125	0.141	Synergism
Oritavancin	8	4.0	0.25		
Cefoxitin	4	1.0	0.25	0.5	Synergism
Oritavancin	8	4.0	0.25		
Moxifloxacin	1	0.25	0.25	0.5	Synergism
Oritavancin	8	0.125	0.0156		
Meropenem	4	1.0	0.25	0.266	Synergism
Oritavancin	8	1.0	0.125		
Levoxofloxacin	2	1.0	0.5	0. 563	No interaction
Oritavancin	8	0.5	0.0625	0.508	
Amikacin	8	4.0	0.5		No interaction
Oritavancin	8	2.0	0.125	0.625	
Bedaquiline	0.25	0.125	0.5		No interaction
Oritavancin	8	8	0.5	1.0	
Linezolid	0.25	0.125	0.5		No interaction
Oritavancin	8	0.125	0.0078	1.01	
Rifampicin	1	1.0	1.0		No interaction
Oritavancin	8	8.0	0.5	1.0	
Imipenem	16	8.0	0.5	0.5	No interaction

## Data Availability

Data sharing not applicable. No new data were created or analyzed in this study. Data sharing is not applicable to this article.
